# Staged surgery for the treatment of carotid aneurysm

**DOI:** 10.1093/jscr/rjae849

**Published:** 2025-01-14

**Authors:** Shiyi Zhao, Dejie Chen

**Affiliations:** College of Medicine, Wuhan University of Science & Technology, Wuhan 430081, China; Department of Vascular Surgery, Xiangyang Central Hospital, Affiliated Hospital of Hubei University of Arts and Science, Xiangyang 441021, China

**Keywords:** carotid artery block, staged surgery, carotid aneurysm

## Abstract

Extracranial carotid artery aneurysm (ECAA) is a relatively rare vascular lesion of the neck, and is usually found incidentally and is usually asymptomatic. Surgery is currently the first choice for symptomatic or growing ECAA, including open resection of the entire aneurysm, with or without arterial replacement and insertion of grafts. Ischemic stroke is the most serious complication after resection of ECAA. The preoperative Matas test facilitates the collateral circulation through the circle of Willis， which allows the brain to adapt to the hypoxic situation and effectively reduces ischemic stroke. We report a case of a young patient who underwent a staged surgery to treat it (the first stage was prophylactic carotid artery blockade, so called open Matas test), and achieved good results.

## Introduction

Extracranial carotid artery aneurysm (ECAA) can lead to rupture of the aneurysm which is a life-threatening condition, and as the aneurysm grows in size it can also press on other organs or even lead to a cerebral infarction [1]. Open resection of the entire aneurysm is currently the first choice for symptomatic or growing ECAA [2, 3]. Ischemic stroke is the most serious complication after resection of ECAA. The Matas test was recommended for the pre-operation preparation [4]. In young patients with an incomplete circle of Willis and aneurysmal anatomical characteristics that contraindicate endovascular repair, or when prolonged cerebral ischemia is anticipated during surgery due to procedural complexity, performing the Matas test is recommended to facilitate cerebral adaptation to hypoxic conditions. However, if the Matas test is conducted using conventional techniques, it may not effectively reduce the risk of ischemic stroke. In 2024, a young patient with ECAA in our institution has received a staged surgery to treat it (the first stage was prophylactic carotid artery blockade, so called open Matas test), and achieved good results, which is reported as follows. Patient has given consent to publish case images.

## Case report

**Figure 1:**
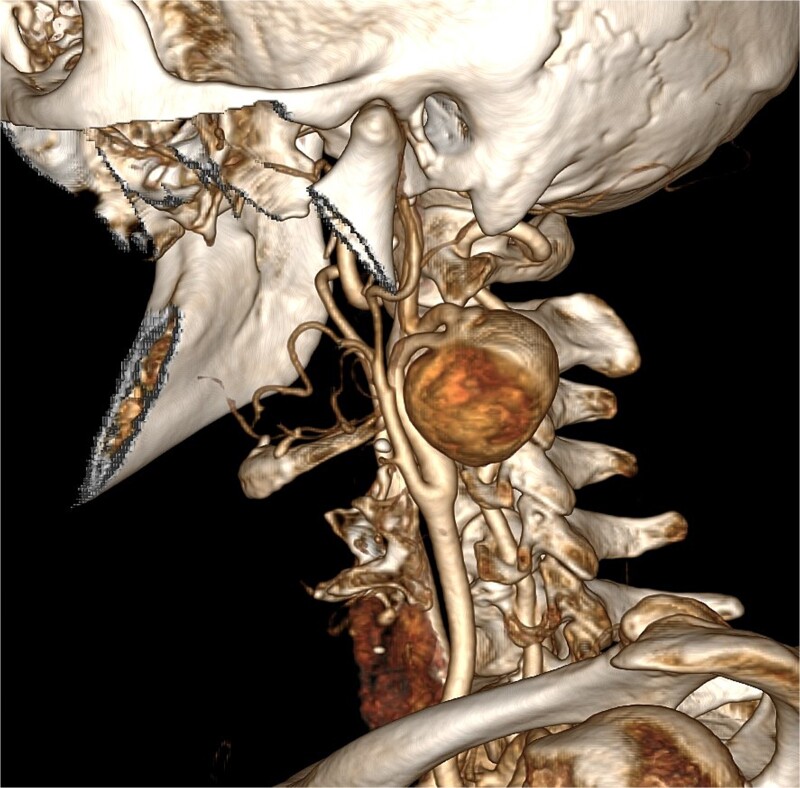
The carotid aneurysm located at the internal carotid artery and compressed the C1 segment, emanated from the distal end of the C1 segment at an angle of 90°, and the distal end of it formed three more 90° angles then traveled upward to the C2 segment. The anatomical characteristics of the aneurysm indicated that the endovascular repair was impossible.

We present the case of a 45-year-old woman who incidentally found a mass in her left neck 6 days ago. After hospitalized, the computed tomography angiography (CTA) of neck and head was performed, showed an aneurysm from C1 to C2 segment of the internal carotid artery and incomplete circle of Willis (Fig. 1). The anatomical characteristics of the aneurysm indicated that the endovascular repair was impossible.

**Figure 2:**
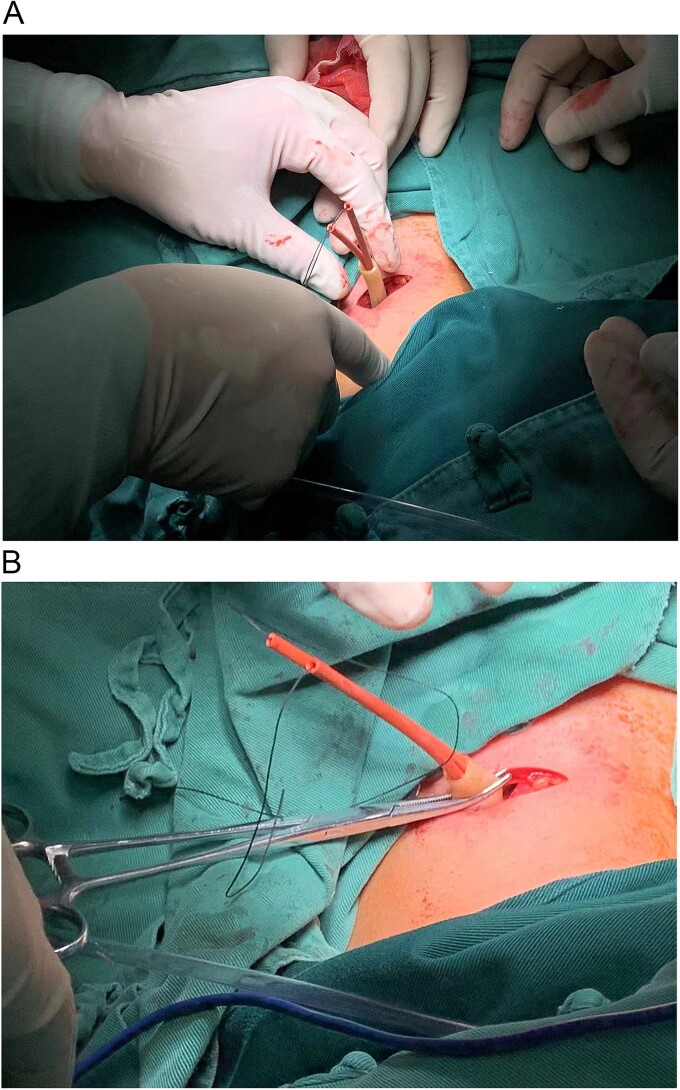
(A) Following routine disinfection and sterile draping, local anesthesia was injected, a transverse 3 cm incision was made ⁓2 cm above the sternal notch along the dermatome of the left neck. After the exposure of the left common carotid artery, an 8-French catheter was looped around the common carotid artery, with the tail of the catheter passed through the incision in a vertical orientation. (B) A short section of the T-tube was attached to the external part of the catheter and secured to prevent loosening. Upon tightening the catheter, aneurysm pulsation ceased, indicating successful blockage, and pulsation returned upon loosening the catheter, the platysma and skin were sutured.

The patient was young with incomplete circle of Willis and expected long time cerebral ischemia during surgery, all of these increased the risk of ischemic stroke both during and after the operation. So we chose an open carotid artery block surgery rather than traditional Matas Test to ensure the brain to adapt to the hypoxic situation. The operation lasted 30 min, with an estimated blood loss of 10 ml. Surgical outcomes are shown in Fig. 2.

Postoperatively, the wound, catheter, and T-tube were covered with sterile dressings. The Patient began functional carotid artery blockade exercises on the same day after surgery. Before each Matas test, the skin within 10–15 cm around the wound and the T-tube were thoroughly sterilized. The catheter was lifted upward, and the T-tube was pushed downward until the aneurysm pulse disappeared, confirming effective carotid blockade. The catheter was then secured with sterile vascular clamps, which were removed at the end of the exercise. The T-tube was repositioned upward to ensure the return of the aneurysm pulse. After each session, the wound, catheter, and T-tube were sterilized again and re-covered with sterile dressings. The exercise was performed 3–5 times per day, with blocking time gradually increased from 5 min to 10 min, then 15 min, depending on the patient’s tolerance, with a target duration of 30 min. The exercise was immediately halted if the patient experienced dizziness, headache, or other symptoms. The exercise lasted 10 days, during which the patient’s maximum tolerated blocking time reached 1 h.

**Figure 3:**
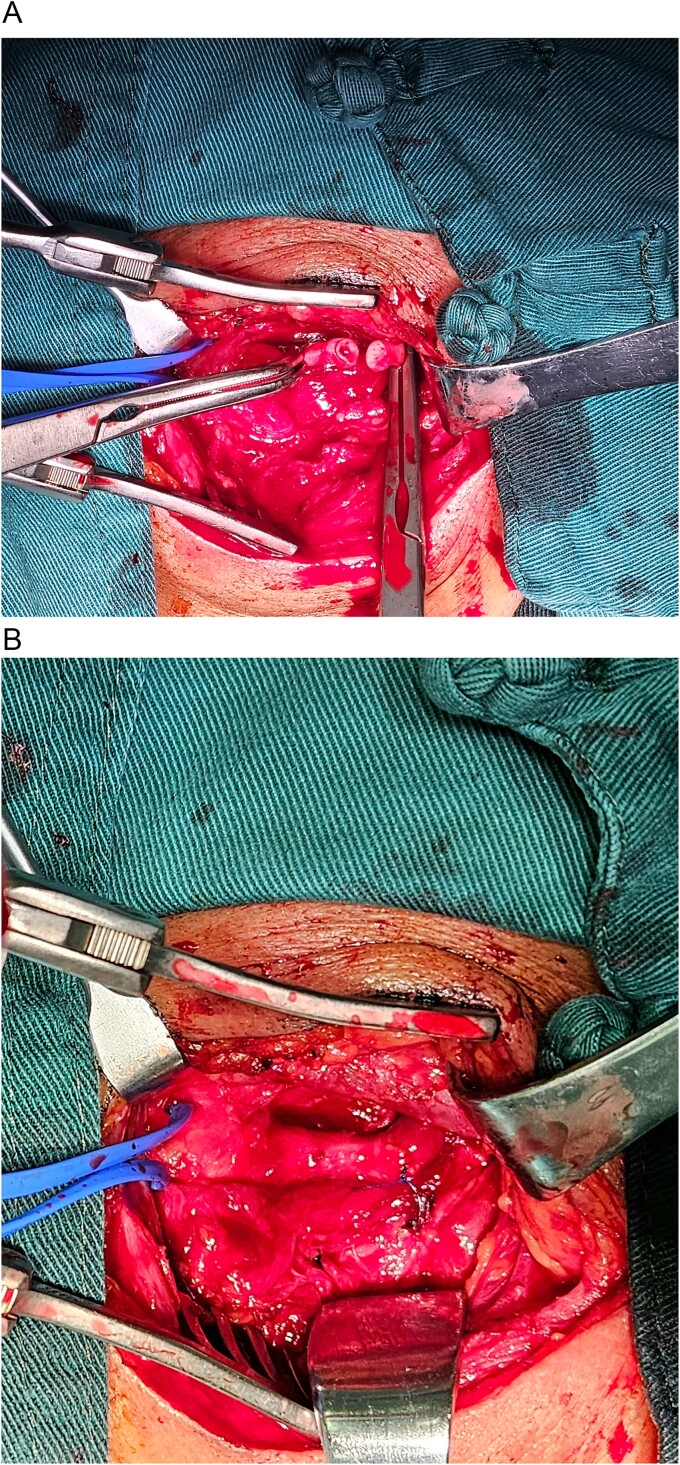
(A) After systemic heparinization with systemic heparin injection and clamping of the distal C1 and proximal C2 segments, the aneurysm was completely resected, resulting in a successful anastomosis. (B) An in situ internal carotid artery-to-internal carotid artery anastomosis was performed, demonstrating no blood leakage and strong distal pulsation.

On the 10th day after the first procedure, resection of the left internal carotid artery aneurysm with in situ internal carotid artery reconstruction was performed under general anesthesia. Noting that the initial surgical incision appeared fresh and infection-free, the T-tube and urinary catheter were removed before covering the area with a sterile protective film. A 7 cm longitudinal incision was made along the anterior edge of the sternocleidomastoid muscle. Dissection was extended to expose the carotid artery bifurcation and C1 segment, allowing for complete aneurysm exposure. The C2 segment of the internal carotid artery was freed 2 cm distal to the aneurysm. Care was taken throughout the procedure to protect the internal jugular vein, vagus nerve, hypoglossal nerve, and other critical structures until the full aneurysm was revealed. The aneurysm was completely resected, resulting in a successful anastomosis (Fig. 3). Once hemostasis was achieved, a negative pressure drain was placed, and the wound was closed in layers with absorbable sutures. The intraoperative cerebral ischemia time was ⁓40 min, with an estimated blood loss of 50 ml.

The drainage tube was removed on the third postoperative day, and the patient was discharged from the hospital on the fifth day without any complaints of abnormal symptoms. One month later, a follow-up carotid CTA revealed the absence of the carotid aneurysm and normal blood flow through the internal and external carotid arteries.

## Discussion

ECAA often presents as an asymptomatic, slow-growing neck mass [1]. If not diagnosed and treated promptly, it can lead to serious complications, including cerebral infarction, compression of surroundings structures or aneurysm rupture, and rarely up to death [3, 5]. Open repair of the aneurysm is currently the first choice for symptomatic or growing ECAA, and endovascular repair of it was also reported in recent years. In this case, the anatomical characteristics and the size of aneurysm demonstrated that open repair was the only way to treat it [6, 7].

In this case, the aneurysm needed a direct resection and an end-to-end arterial anastomosis. This young patient with an incomplete circle of Willis and expected a prolonged cerebral ischemia indicated high risk of ischemic stroke. Several studies have demonstrated that Matas training—based on the principle of promoting cerebral collateral circulation via unilateral carotid artery compression—facilitates cerebral adaptation to extended ischemic and hypoxic condition, reduces intraoperative risk of ischemic stroke [4, 8]. However, the traditional Matas training is performed by patients under the guidance of healthcare personnel, due to the patient’s unfamiliarity with the carotid anatomy, it is difficult for them to keep the finger immobile for a longer period of time during compression, and prolonged compression can easily lead to weakening and stiffening of the patient’s fingers, which cannot ensure the carotid arteries remain in a state of complete blockage, also easily result in the displacement of the position of the compression. Therefore, it is not possible to accurately assess the effective duration of carotid artery blockade or the duration of tolerance of unilateral cerebral ischemia after carotid artery blockade, which greatly increases the risk of ischemic stroke in patients during and after surgery.

We provided a new idea to this confusion for the treatment of complicated ECAA. The first stage to ensure the duration of tolerance of unilateral cerebral ischemia after carotid artery blockade was performed, which greatly decreases the risk of ischemic stroke in patients during the second stage. Meanwhile, during the second procedure, surgeons who performed the procedure had abundant time to expose aneurysm, which could reduce the risk of neural and vascular injury, obtained a better quality anastomosis at state of relatively relaxed mind.

Compared with the conventional approach, the drawback of this method lies in the necessity for two separate procedures, resulting in increased procedural trauma. However, considering the risk of cerebrovascular events, the additional trauma is justifiable, particularly in younger patients.

## Conclusions

Staged surgical intervention provides an alternative treatment option for young patients with carotid artery aneurysms, particularly those with an incomplete circle of Willis and anatomical contraindications to endovascular repair. Although the risk of infection persists throughout the procedure, it can be effectively mitigated through stringent aseptic techniques at every stage.
